# Soluble angiotensin-converting enzyme 2 association with lipid metabolism

**DOI:** 10.3389/fmed.2022.955928

**Published:** 2022-08-10

**Authors:** Izumi Nagatomo, Kaori Nakanishi, Ryohei Yamamoto, Seiko Ide, Chisaki Ishibashi, Toshiki Moriyama, Keiko Yamauchi-Takihara

**Affiliations:** Health Care Division, Health and Counseling Center, Osaka University, Osaka, Japan

**Keywords:** sACE2, obesity, total cholesterol, triglycerides, COVID-19

## Abstract

Increased expression of angiotensin-converting enzyme 2 (ACE2) is one of the likely explanations for disease severity in patients with coronavirus disease 2019 (COVID-19). In this study, we aimed to test whether soluble ACE2 (sACE2) levels are correlated to known risk factors of severe COVID-19 including biochemical parameters, body mass index and smoking habits. We cross-sectionally evaluated serum sACE2 levels in obese or tobacco-smoking populations and compared them to those in non-obese and non-smoking healthy participants. Additionally, fibroblast growth factor-21 (FGF21) was investigated as a candidate regulator of sACE2. A total of 220 male participants aged 30–59 years undergoing an annual health checkup were enrolled in this study: 59 obese, 80 smokers, and 81 healthy. Serum sACE2 levels were significantly higher in obese participants but not in tobacco-smoking participants when compared to healthy participants. sACE2 levels were significantly correlated with total cholesterol and triglycerides but not with body mass index. Furthermore, no regulatory relationship was found between FGF21 and sACE2. Lipid metabolism disorders accompanied by upregulation of serum sACE2 may be underlying mechanisms of COVID-19 aggravation and might be a novel breakthrough treatment target.

## Introduction

The severity of coronavirus disease 2019 (COVID-19) depends on comorbidities; diabetes mellitus (1), hypertension (2), dyslipidemia (3), metabolic syndrome (4), obesity (5), age (6), male sex (7), and smoking habits (8) are known risk factors for aggravation of the disease. However, the causative relationships between comorbidities and disease severity remain unclear. Angiotensin-converting enzyme 2 (ACE2), which is expressed in various tissues, is an important regulator of the renin-angiotensin-aldosterone system and has attracted much attention as a receptor for the coronavirus (9–11). Increased ACE2 expression may be a potential cause of aggravation because it can lead to higher amounts of viral entry into target cells (12). Previous studies have shown a relationship between ACE2 expression levels and some comorbidities; for example, hyperglycemia increases monocyte surface ACE2 expression (13). Smoking has also been shown to upregulate ACE2 expression in the small airway epithelium and pneumocytes (14).

However, assessment of tissue ACE2 expression is technically difficult; therefore, soluble ACE2 (sACE2) in the serum has been evaluated as an alternative. Serum sACE2 has been reported to be upregulated in individuals with obesity and lifestyle-related diseases (15, 16). ACE2 and serum sACE2 expression are also known to be higher in men than in women (17). These findings suggest that higher ACE2 expression could explain the higher risk of severe COVID-19 in patients with these various backgrounds. However, it remains unclear how ACE2 expression is regulated in normal and diseased individuals.

Fibroblast growth factor-21 (FGF21) is a type of cytokine involved in metabolism and is also classified as a type of mitokine that is produced in response to mitochondrial stress (18). Serum levels of FGF21 are increased in individuals with obesity, type 2 diabetes, and metabolic syndromes (19, 20). We also previously reported that smoking increased the serum levels of FGF21 (21). Of note, FGF21 was reported to induce ACE2 expression in adipocytes and renal cells in mouse models (22), which led to our hypothesis that FGF21 upregulation might cause aggravation of COVID-19 *via* induction of ACE2 in various lifestyle-related diseases. In the present study, we aimed to test this hypothesis by performing a series of cross-sectional examinations focusing on serum sACE2 and FGF21 levels.

## Methods

### Study participants

This study contains cross-sectional data obtained from employees of Osaka University. The participants were selected from male examinees aged 30–59 years undergoing an annual health checkup at the Osaka University Health and Counseling Center. Patients with chronic illness or taking regular medication for at least 1 year before their health checkup were excluded in advance.

Participants were enrolled with a goal of roughly 300 cases, including obese, smoking, and healthy groups. For the obese group, examinees with a body mass index (BMI) of 30 or higher were selected, and current smokers were excluded from the obese group to avoid complexity. For the smoking group, currently smoking examinees were selected from those with a Brinkman Index of 100 or higher. As a result, there were no current smokers in the obese group, and none in the smoking group met the obesity criteria. As normal participants, healthy examinees were randomly selected who met the following conditions: blood pressure (BP) less than 140/90 mmHg, BMI 18.5–25 kg/m^2^, hemoglobin A1c (HbA1c) less than 5.6%, and non-smokers.

This study was performed in accordance with the Declaration of Helsinki and the Ethics Guidelines for Clinical Research from the Ministry of Health, Labor and Welfare, and the Ministry of Education, Culture, Sports, Science and Technology. All experimental protocols in this study were approved by the Ethics Committee of Health and Counseling Center, Osaka University, and written informed consent was obtained from all participants prior to participation in the study.

### Assessments of physical and biochemical parameters

Body mass index and systolic and diastolic BP were measured as physical parameters. Information on participants’ medical history, current treatments, smoking status, and lifestyle behavior was obtained *via* questionnaires, which was reconfirmed through expert interviews by trained nurses.

Serum samples were collected from participants in the morning after an overnight fast and kept at ≤−20°C until assayed. Serum concentrations of aspartate aminotransferase (AST), alanine aminotransferase (ALT), gamma-glutamyl transpeptidase (γ-GTP), creatinine (Cr), uric acid (UA), total cholesterol (TCHO), triglyceride (TGL), high-density lipoprotein cholesterol (HDL), low-density lipoprotein cholesterol (LDL), glucose (Glu), and HbA1c were measured as part of an annual health checkup. For this study, serum levels of sACE2 and FGF21 were measured using the surplus of the same serum samples as described above. Serum concentrations of FGF21 were measured using a sandwich enzyme-linked immunoassay system according to the manufacturer’s instructions (R&D Systems Inc., Minneapolis, MN, United States). Serum concentrations of sACE2 were also measured using a sandwich enzyme-linked immunoassay system according to the manufacturer’s instructions (BioVision, Milpitas, CA, United States).

### Statistical analyses

All statistical analyses were performed using STATA 16 (STATA Corp LLC, College Station, TX, United States). The distribution of continuous variables was tested using the Shapiro–Wilk test. Normally distributed variables are presented as means ± SD; non-normally distributed variables are presented as medians with interquartile ranges. The ANOVA followed by Tukey-Kramer pairwise comparison, or the Kruskal–Wallis equality-of-populations rank test, followed by Dunn’s pairwise comparison, was used to test differences between the three groups. The Spearman’s rank correlation coefficient was used to analyze the variables. All *p*-values were two-sided, and a *p*-value < 0.05 was considered statistically significant.

## Results

### Age dependency of serum soluble angiotensin-converting enzyme 2 levels

There were several cases in which the surplus samples were insufficient and could not be measured. An additional 16 participants were excluded because serum sACE2 or FGF21 were out of the measurable range and no precise data were available. As a result, a total of 220 men were enrolled in this study: 59 obese, 80 smokers, and 81 healthy.

First, all study participants were divided into three groups according to age, as follows: 30–39, 40–49, and 50–59 years (Table 1). Serum sACE2 levels showed no significant differences with age. On the other hand, FGF21 levels significantly increased with age, consistent with our previous reports (23). BMI was significantly lower in the 30–39 age group.

**TABLE 1:** Characteristics by age group.

Age group	30–39	40–49	50–59
	(*n* = 108)	(*n* = 66)	(*n* = 46)
BMI (Kg/m^2^)	22.9 (21.0–24.7)**^a,^ **^b^	24.3 (21.6–30.6)**^a^	24.6 (22.7–30.2)**^b^
sACE2 (pg/ml)	71.1 (51.7–87.3)	64.9 (46.9–89.3)	68.7 (52.2–99.3)
FGF21 (pg/ml)	123.5 (79.7–211)**^b,^ *^a^	155 (107–215)*^a,^ **^c^	207 (151–443)**^b,^ **^c^

Data are expressed as medians (interquartile range). The Kruskal–Wallis equality-of-populations rank test followed by Dunn’s pairwise comparison was used to test between-group differences.

*p < 0.05; **p < 0.01.

^a^30–39 vs. 40–49, ^b^30–39 vs. 50–59, ^c^40–49 vs. 50–59.

BMI, body mass index; sACE2, soluble angiotensin-converting enzyme 2; FGF21, fibroblast growth factor 21.

### Soluble angiotensin-converting enzyme 2 levels in obese and smoking groups

**FIGURE 1:**
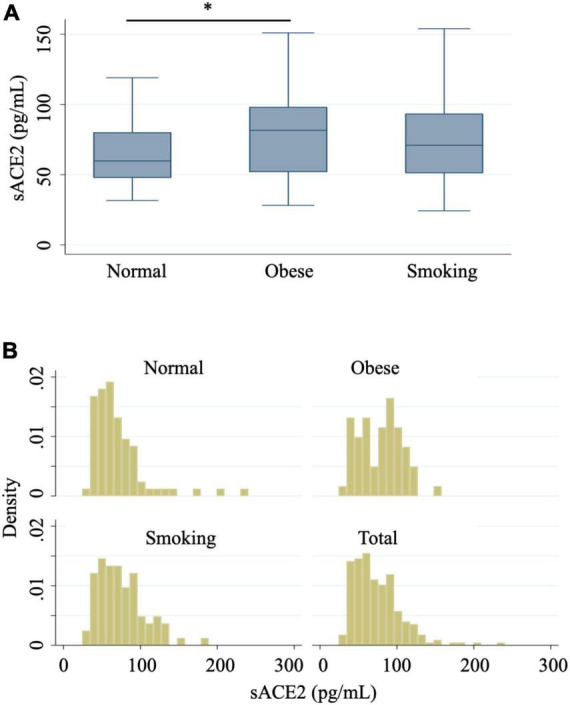
Serum sACE2 levels in normal, obese, and smoking groups. **(A)** Box plots show serum sACE2 levels from normal, obese, and smoking groups. The Kruskal–Wallis equality-of-populations rank test, followed by Dunn’s pairwise comparison, was used to test between-group differences. *p < 0.05. **(B)** Histograms provide a visual representation of data distribution in serum sACE2 levels from normal, obese, smoking, and total groups.

Next, we investigated whether serum sACE2 levels were altered in the obese and smoking groups compared to the normal group. Serum sACE2 levels were significantly increased in the obese group compared to those in the normal group (Figure 1A). On the other hand, serum sACE2 levels were not significantly different between the smoking and normal groups (Figure 1A). The median serum sACE2 levels were 59.8, 81.6, and 70.9 pg/ml in the normal, obese, and smoking groups, respectively. As shown in Figure 1B, data distribution displayed the difference between obese and other groups, suggesting the upregulation of serum sACE2 in obesity.

### Association of soluble angiotensin-converting enzyme 2 levels with metabolic parameters

To further elucidate the role of serum sACE2 in obesity and related systemic metabolic disorders, various parameters, including FGF21, were analyzed. The characteristics of the three groups (normal, obese, and smoking) are shown in Table 2. As expected, TCHO and TGL were significantly higher in the obese and smoking groups compared to the normal group (Table 2). Differences between the obese and smoking groups depended on the parameters.

**TABLE 2:** Comparison of characteristics between normal, obese, and smoking groups.

	Normal	Obese	Smoking
	(*n* = 81)	(*n* = 59)	(*n* = 80)
Age (years)	36 (32–42)^a, b^	43 (36–49)^a^	44 (34.5–51.5)^b^
BMI (Kg/m^2^)	22 (20.1–23.2)^a, b^	31.3 (30.4–33)^a, c^	23.2 (20.9–24.65)^b, c^
sBP (mmHg)	115.12 ± 12.04^a, b^	131.61 ± 11.37^a, c^	121.09 ± 14.03^b, c^
dBP (mmHg)	70.41 ± 7.85^a, b^	86.42 ± 10.44^a, c^	77.5 ± 10.53^b, c^
AST (IU/L)	18 (16–20)^a, b^	28 (23–37)^a, c^	20.5 (18–25)^b, c^
ALT (IU/L)	17 (13–22)^a, b^	41 (30–70)^a, c^	19.5 (16–29)^b, c^
γGTP (IU/L)	21 (16–31)^a, b^	53 (33–91)^a, c^	28.5 (19–64.5)^b, c^
Cr (mg/dL)	0.88 (0.85–0.95)^b^	0.86 (0.81–0.92)^c^	0.81 (0.75–0.90)^b, c^
UA (mg/dL)	5.76 ± 1.11^a^	6.70 ± 1.10^a, c^	5.91 ± 1.10^c^
TCHO (mg/dL)	187.81 ± 26.76^a, b^	209.59 ± 29.13^a^	200.11 ± 32.10^b^
TGL (mg/dL)	77 (54–112)^a, b^	137 (102–177)^a, c^	93.5 (69.5–132)^b, c^
HDL (mg/dL)	56 (49–63)^a^	48 (42–53)^a, c^	56 (49–68)^c^
LDL (mg/dL)	113.37 ± 24.46^a^	136.42 ± 28.96^a, c^	121.88 ± 30.94^c^
Glu (mg/dL)	84.10 ± 6.06^a^	96.37 ± 26.48^a, c^	87.75 ± 11.48^c^
HbA1c (%)	5.05 ± 0.23^a^	5.62 ± 0.87^a, c^	5.23 ± 0.41^c^
FGF21 (pg/ml)	126 (76.7–187)^a, b^	165 (119–269)^a^	159.5 (104–250.5)^b^

Data are expressed as the mean ± SD or median (interquartile range). The ANOVA followed by Tukey–Kramer pairwise comparison or the Kruskal–Wallis equality-of-populations rank test followed by Dunn’s pairwise comparison was used to test between-group differences.

^a^p < 0.05 (normal vs. obese).

^b^p < 0.05 (normal vs. smoking).

^c^p < 0.05 (obese vs. smoking).

BMI, body mass index; sBP, systolic blood pressure; dBP, diastolic blood pressure; AST, aspartate aminotransferase; ALT, alanine aminotransferase; γ-GTP, gamma-glutamyl transpeptidase; Cr, creatinine; UA, uric acid; TCHO, total cholesterol; TGL, triglycerides; HDL, high-density lipoprotein cholesterol; LDL, low-density lipoprotein cholesterol; Glu, glucose; HbA1c, hemoglobin A1c; FGF21, fibroblast growth factor 21.

Next, we assessed the relationship between serum sACE2 levels and some of the above parameters, confirming that serum sACE2 levels correlated with lipid parameters, TCHO and TGL, but not with Glu (Table 3). However, BMI itself was not significantly correlated with serum sACE2 levels (Table 3). Also, FGF21 did not correlate with serum sACE2 levels (Table 3), suggesting that other mechanisms regulate serum sACE2 levels in these participants. Taken together, serum sACE2 levels certainly increased in obese participants and were more closely correlated to hyperlipidemia than BMI itself in the combined normal, obese and smoking participants.

**TABLE 3:** Correlation of sACE2 and other factors in all the participants.

	Spearman’s rho	*P*-value
Age	0.0032	0.9623
BMI	0.1306	0.053
TCHO	0.1647	0.0144*
TGL	0.2108	0.0017**
Glu	0.058	0.3918
FGF21	0.0287	0.6719

Spearman’s rank correlation coefficient was used to analyze the variables.

*p < 0.05; **p < 0.01.

BMI, body mass index; TCHO, total cholesterol; TGL, triglycerides; Glu, glucose; FGF21, fibroblast growth factor 21.

## Discussion

This study was conducted under the hypothesis that ACE2 expression levels might contribute to COVID-19 aggravation in relation to viral load. We attempted to test this hypothesis by measuring sACE2 at ordinary condition in high-risk populations. We evaluated serum sACE2 levels in obese or smoking individuals and analyzed their relationship with biochemical parameters including FGF21. We found that serum sACE2 levels were significantly elevated in the obese population compared to those in the healthy population. Serum sACE2 levels significantly correlated with TCHO and TGL in all the populations. No correlation was found with FGF21 in any population.

In a previous study, ACE2 expression was found to be upregulated in lung alveolar epithelial cells and adipose tissue in obese or smoking participants (24). These are the target organs of COVID-19, and it is possible that increased expression of ACE2 in these tissues results in increased viral load, contributing to the aggravation of the disease. On the other hand, cytokine storms have been identified as the central pathological mechanism of COVID-19 aggravation (25, 26). In addition, thrombotic tendency is a characteristic of the pathological condition, which is also involved in disease severity (27).

Dyslipidemia is a risk factor for severe COVID-19, probably due to a thrombotic tendency or underlying chronic mild inflammation, which is compatible with our hypothesis in this study. We speculate that the relationship between serum sACE2 levels and lipid metabolism shown in this study also reinforces these findings directly or indirectly. However, the mechanism seems to be complex, and it is certainly not explained by a single factor, such as FGF21. A previous report describes temporal changes in sACE2 associated with metabolic parameters during a weight-loss diet intervention (28). In that study, sACE2 decreased during the intervention and was associated with improvements in metabolic health, including decrease in TGL. Taken together, lipid metabolism is suggested to be intrinsically interacted with sACE2 regulation.

Conflicting reports have been made on the involvement of sACE in the aggravation of COVID-19. We performed this study on the premise that high expression of ACE2 promotes aggravation from the viewpoint of viral load. Indeed, not only ACE2 but also sACE2 can function as a receptor for SARS-CoV-2 (29). However, from the viewpoint of an enzyme, the cardiovascular protective action of ACE2 has been reported to suppress the aggravation. We also assume that sACE2 levels reflect tissue ACE2 expression, but the activity of enzymes such as ADAM17 critically regulates sACE2 levels (30). There are also reports that ACE-I and ARB as antihypertensive drugs do not affect the severity of the disease, making the situation chaotic (31). In addition, our result that sACE2 does not increase in smokers is different from a previous report (15). This difference may be the result of differences in patient background and differences in smoking index standards.

This study has some limitations that should be interpreted with caution. First, from a statistical point of view, the sample size may be insufficient to verify the relationship between some parameters. Second, due to the age bias across all participants, background factors and the obtained data might be biased. Finally, serum sACE2 levels are not necessarily proportional to the ACE2 expression levels in tissues or organs. Despite these restrictions, our data indicate that high expression of ACE2 accompanied by dyslipidemia could be involved in the aggravation of COVID-19. Further investigation is required to fully clarify the significance of serum sACE2 levels in relation to COVID-19 aggravation risk in the obese population.

## Data availability statement

The datasets generated and/or analyzed during this study are available from the corresponding author upon reasonable request.

## Ethics statement

The studies involving human participants were reviewed and approved by Osaka University Health and Counseling Center Research Ethics Review Committee. Written informed consent was obtained from all participants prior to participation in this study.

## Author contributions

IN, KN, and KY-T designed the study. IN performed the experiments, analyzed the data, and wrote the manuscript. CI, KN, and KY-T provided suggestions in the process of this study. SI, RY, and TM provided comments on the first draft. All authors have given approval to the final version of the manuscript.
